# Are urban residents seeking appropriate care for malaria? evidence from an exploratory qualitative study in two cities in nigeria

**DOI:** 10.1186/s12913-024-12013-9

**Published:** 2024-12-18

**Authors:** Akintayo Ogunwale, IkeOluwapo Ajayi, Eniola Bamgboye, Al-Mukhtar Adamu, Musa Bello, Morenikeji Olawuwo, Adeniyi Fagbamigbe, Joshua Akinyemi, Ifeoma Ozodiegwu

**Affiliations:** 1 Department of Public Health, College of Health Sciences, Bowen University, Iwo, Osun Nigeria; 2 Epidemiology and Biostatistics Research Unit, Institute for Advanced Medical Research and Training (IAMRAT), College of Medicine, University of Ibadan, Ibadan, Oyo Nigeria; 3 Department of Epidemiology and Medical Statistics, Faculty of Public Health, College of Medicine, University of Ibadan, Ibadan, Oyo Nigeria; 4 Department of Medical Microbiology and Parasitology, Bayero University, Kano, Nigeria; 5 Department of Community Medicine, Bayero University, Kano, Nigeria; 6 Department of Preventive Medicine and Institute for Global Health, North-western University, Chicago, IL USA; 7 Department of Health Informatics and Data Science, Loyola University Chicago, Health Sciences Campus, Maywood, IL 60153 USA

**Keywords:** Malaria, Urbanization, Healthcare providers, Nigeria

## Abstract

**Background:**

Unplanned and rapid urbanization within Nigerian cities with the attendant environmental consequences may hinder achieving malaria elimination goal. Presently, there are limited qualitative studies on malaria case management and care-seeking patterns by settlement type in urban areas in Nigeria. This study, investigated malaria-related health seeking behaviours among different settlement types in Ibadan and Kano metropolises, Nigeria.

**Methods:**

This qualitative study involved conduct of nine Focus Group Discussions (FGDs) sessions among community members and 20 Key Informant interviews (KIIs) with healthcare providers and community stakeholders in each metropolis. Participants were purposively drawn from three settlement types – formal, informal and urban slum. Pretested FGD and KII guides were used to collect data. Data were subjected to thematic content analysis.

**Results:**

Government-owned health facilities especially Primary Health Care (PHC) facilities was a major place identified as where many community members prefer to seek care for suspected malaria infection. Reasons adduced included proximity of facilities and services affordability. A common viewpoint was that most community members in informal and slum communities in both metropolises often patronize Proprietary Patent Medicine Vendors (PPMVs) or drug sellers as the first point for treatment of suspected malaria infection. Adduced reasons included poverty and non-availability of 24 h services in PHC facilities. High cost of treatment, poor attitude of health workers, long waiting time and cultural beliefs were identified as key factors influencing community members’ decision not to seek care in health facilities. It was noted that the rich prefer to seek treatment in private hospitals, while the poor usually visit PHCs or resort to other options like buying drugs from PPMVs or use herbal drugs.

**Conclusion:**

The standard practice of seeking care in health facilities was influenced by diverse factors including treatment cost, health workers’ attitude and waiting time in health facilities. A commoner practice of seeking care from patent medicine vendors and herbal drug sellers among informal settlements and slums dwellers calls for public health interventions. Specifically, the provision of free or affordable malaria care in health facilities and patient-friendly environments; and training of non-formal care providers on appropriate malaria care and referral to health facilities are recommended.

**Supplementary Information:**

The online version contains supplementary material available at 10.1186/s12913-024-12013-9.

## Background

Malaria is still a major global public health concern, despite frantic efforts and interventions targeted at its elimination. Malaria disease can be categorized based on degree of severity into uncomplicated or severe (complicated). In general, malaria is a curable disease condition if diagnosed and treated promptly and correctly. The vast majority of malaria cases and deaths are found in Africa, with nearly all cases caused by the *Plasmodium falciparum* parasite [1]. Nigeria alone accounts for more than one-quarter (27.0%) of all the malaria deaths worldwide in 2023 [2].

Malaria accounts for the highest number of hospitalizations and outpatient visits in Nigeria [1, 3, 4]. The most vulnerable Nigerians are under-five children, who experience an average of 2–4 episodes per year and account for as much as 90% of national malaria mortality [5]. One of the major contributors to the global malaria burden especially in malaria endemic countries like Nigeria is the rapid unplanned urbanization with the attendant challenges of overcrowding and environmental degradation, leading to growing concerns that malaria transmission could increase substantially in cities [2, 6].

Appropriate and prompt health-seeking behaviour for malaria treatment is fundamental in the success of morbidity and mortality prevention strategies [5, 7]. Health seeking behaviour, situated within the broader concept of health behaviour, can be defined as, action or inaction undertaken by individuals who perceive themselves to have a health problem or to be ill for the purpose of finding an appropriate remedy or care [8]. Some decisions taken after the onset of symptoms presumed to be malaria include staying home and doing nothing, treatment with herbal medication, self-medication with over-the-counter drugs, and visits to the health facilities [7]. Fever is the most common symptom for malaria, but unfortunately shares commonality with other febrile illnesses that are also prevalent in malaria endemic areas [9, 10]. It is, therefore, pertinent that persons with fever should seek appropriate care to rule out malaria among other causes of febrile illness [9].

Nigeria remains one of the countries with low rates of care-seeking for suspected malaria cases among under-five children [2, 11]. This is largely attributable to a health financing system that leaves many individuals uninsured, resulting in high out-of-pocket medical expenditure that discourages care-seeking, especially among the poor [12]. The 2021 Nigeria Malaria Indicator Survey [13] as well as some other previous studies [9, 10] reported that malaria care-seeking and behaviours vary across and within regions and settings including urban areas in Nigeria.

While there is extensive literature, especially sentinel studies pointing out the burden of malaria and inappropriate malaria care-seeking behaviours in several urban areas in Nigeria [3, 8, 14, 15], there are almost no studies on malaria care-seeking behaviour focusing on city-level variations along settlements types. Additionally, most of the available sentinel studies such as [8, 13, 14] did not capture the malaria care-seeking behaviour patterns of urban residents across different regions in the country.

Pertinently, qualitative studies providing deeper understanding of the behaviours relating to health care seeking on malaria are needed in the country. Robust evidence-based information from qualitative studies on malaria-related health seeking behaviours of community members in different settlement types in large urban cities like Ibadan and Kano is important and can help to inform policies, programme implementation as well as provide evidence-based insights for targeted intervention for the different settlement types within urban areas. This study was designed to investigate the malaria-related health seeking behaviours among community and healthcare stakeholders residing in Ibadan and Kano metropolises. The specific objectives of the study were to: assess opinions about where community members seek care for suspected malaria infection; identify the factors influencing the choice of health facility where community members seek care for suspected malaria infection; describe the perceptions relating to the differences in health seeking for suspected malaria infection by socio-economic groups; assess opinions about the appropriate sources of health care for malaria.

## Methods

This work formed part of the first phase of a project funded by Bill and Melinda Gates Foundation to assess the burden and determinants of malaria transmission for tailoring of interventions (microstratification) in Ibadan and Kano metropolises. The study was conducted among adult community members as well as community stakeholders and healthcare providers residing in Ibadan and Kano metropolises between January and March, 2022. The Standards for Reporting Qualitative Research (SRQR) guidelines [16] was followed.

### Study design

The study employed an exploratory qualitative study design which involved Focus Group Discussions (FGDs) and Key Informant Interviews (KIIs).

### Study settings

The study was carried out in two Nigerian urban cities, namely, Ibadan and Kano metropolises. Ibadan is one of the largest cities in West Africa, and is located in the South-west geo-political zone of Nigeria, while Kano is the largest cosmopolitan setting in Northern Nigeria and it is in the North-west geo-political zone of the country. Ibadan currently serves as the capital of Oyo State, while Kano city is the capital of Kano State. Ibadan metropolis area is divided administratively into five LGAs, further subdivided into 59 wards and each has a mean population size of 12, 665 persons (Standard Deviation (SD): 5,802) [17]. Kano metropolis area consists of six LGAs, which are subdivided administratively into 66 wards. The mean population density in Kano metropolis is 23, 401 persons per square kilometre (SD – 17, 313) [17]. The two cities are among Nigeria's fastest growing urban areas with the features of the different settlements’ types, namely: formal settlements, informal settlements and slums. The settlement types (formal, informal and slums) were classified based on the findings from a Multi-Stakeholders Dialogues (MSDs) and defined as follows [18]:

1. Formal settlements were conceptualized as well planned layout areas or government residential areas not densely populated. They are characterised by availability of social amenities and infrastructure including good roads, electricity, good drainage systems, schools, hospitals and police stations.
2. Informal settlements were considered as areas not well developed, basically developed by the community and not by the government consisting of mixed types of houses not following any government regulations. They are characterised with inadequate social amenities and infrastructure.
3. Slums were operationally defined as settlements that are unplanned and have abysmal or poor housing conditions and with no or limited basic amenities and usually densely populated and inhabited by people of low socio-economic status.

Four wards were selected in Ibadan metropolis, namely: Agugu; Olopomewa; Basorun; and Challenge, representing the settlement types, for the study. While in Kano metropolis, five wards (Dorayi, Fagge, Tudun Wazurchi, Gobirawa and Zango) were selected. A combination of strategies including a model-based clustering approach, site visitations and Multi-Stakeholders Dialogue (MSDs) was used to inform the categorization and selection of wards in both metropolises. Extensive description of the study settings as well as the selection of the wards and characteristics of the selected wards in each of the cities has been published [18].

### Sample size and sampling

Nine FGDs were conducted in each of the two metropolises among purposively selected participants drawn from the different settlement types (slums, informal settlements and formal settlements). Specifically, the nine FGDs conducted in each metropolis comprised three male adult groups (one per settlement type); three female adult groups (one per settlement type); and three which targeted mothers of under-five children (one from each settlement type). A total of 20 KIIs targeting various categories of key stakeholders (formal healthcare workers – 10; non-formal healthcare providers—6; community leaders −4) were carried out in each of the two metropolises. In this context of the study, ‘formal healthcare workers’ are healthcare professionals who have formal training and experience in providing healthcare treatment and advice. We operationalized ‘Informal healthcare providers’ (Informal healthcare workers) as caregivers or individuals who provide healthcare services without formal training or regulation in the community. These categories of persons include Proprietary Patent Medicine Vendors (PPMVs), drug sellers and traditional doctors/healers. The FGD participants and key informants were purposively recruited with the support of community gatekeepers identified through a community engagement process. The KIIs and FGDs were conducted up until saturation level was achieved. More detailed information about the sampling and the recruitment have been captured in the protocol paper earlier published [18].

### Data collection

Data were collected using validated FGD and KII guides developed by the research team (see supplementary materials). The guides, consisting of open-ended questions, focused on various issues including: opinions about where community members seek care for suspected malaria infection; factors influencing the choice of health facility where community members seek care for suspected malaria infection; beliefs about the appropriate sources of health care for malaria. A separate KII guide was used for each of the three categories of the key informant interviewees, namely, community stakeholders, formal health workers and informal healthcare providers. The instruments were pretested before use in both study sites among individuals who shared similar characteristics with the study participants before use [18].

Community gatekeepers including Ward Development Committee (WDC) chairs as well as community liaison officers, identified through community engagement process, assisted in mobilizing the participants especially community members for active participation in the study. The conduct of each of the FGDs and KIIs was done by a team of two trained Research Assistants (RAs) comprising a moderator and a note-taker who were familiar with qualitative study and the study terrains.

Five teams were employed in total. The RAs were supervised by field supervisors and project team members. The RAs involved in each study site had a minimum of first degree and at least two years’ experience in conducting qualitative research and were fluent in both English language and the study site local language (Yoruba for those in Ibadan metropolis and Hausa in Kano metropolis).

The KIIs and FGDs were conducted in mutually agreeable and convenient venues like offices, community halls, community square and backyards of residence that were devoid of distraction and factors that could affect free flow of discussion or interview. The venues used had comfortable seats and good ventilation. Each of the KIIs and FGDs was guided by an appropriate guide and tape-recorded after obtaining the consent from the participants. Average time for the KIIs was 30 min, while that of FGDs was 45 min. Each day’s activities ended with the RAs filling a debriefing form (uploaded to google cloud) designed for monitoring the activities on the field.

### Data analysis

Data analysis was carried out using thematic content analysis procedures. The process started with the transcription of the FGDs and KIIs tape recordings that was done few hours after the data were collected to avoid loss or omission of important details. In addition, all the FGDs and KIIs done in local languages (Hausa and Yoruba languages) were expertly forward-back translated to English. All the transcribed notes were further subjected to audit and validation. Two field supervisors and four team members (AO, IA, EB, AA, MB) who were senior academics with extensive experience in qualitative research were involved in the data audit and validation. Thereafter the validated transcribed notes were entered into the computer using NVIVO (version R 1.7 Pro) by the qualitative data analyst (AO). Coding of the data was done using the inductive-dominant coding approach [19]. Additionally, codes were structured, organized, reviewed and merged based on the manifest and latent contents of the data. The codes were linked appropriately to the corresponding quotations. Themes emerged based on patterns of results as reflected in similar codes and quotations. Themes were further explored along the pattern of results based on major points of agreements, contrasting views or points, striking and salient points that were made by the participants. The themes generated were organized in line with the study objectives and reviewed by six project team members (AO, IA, EB, AA, MB, MO, IO). The last procedure involved in the analysis of the data was the writing of summaries supported with appropriate verbatim quotes. Both the FGD and KII data were triangulated.

### Rigour

The use of field notes, reflexive diaries and team reviews of activities and data were some of the strategies employed to promote trustworthiness of the data. We documented an audit trail of all the data collection processes and data analysis throughout the study to ensure confirmability. Extended engagements with participants, triangulation of data sources, close supervision of data collection process and data validations were parts of the methods employed for achieving credibility. Additionally, background information about each of the participants were provided to enhance transferability. The study team including the authors had no relationships with the participants prior to the commencement of the study. Throughout the research, the authors were cautious not to make premature assumptions. We maintained open communication among the authors to encourage thoughtful reflection.

## Results

### Socio-demographic characteristics of FGD participants

The socio-demographic information of the FGD participants is presented on Table 1. A total of 157 persons participated in the FGDs. The proportions of FGD participants who were males and females were 33.8% and 63.2% respectively. The proportions of the FGD participants residing in formal settlements, informal settlements and slums across both metropolises were 31.8%, 43.3% and 24.8% respectively. Majority (43.6%) of the participants had secondary education, while 12.8% had no formal education. The participants were into various occupations including trading (46.4%) and craftwork (18.6%).

**Table 1:** Frequency Distribution of the Socio-demographic characteristics of Focus Group Discussion participants

Variable	Ibadan n (%)	Kano n (%)	Total n (%)
**Sex (*N* = 157)**			
Male	25 (16.0)	28 (17.8)	53 (33.8)
Female	45 (28.6)	59 (37.6)	104 (66.2)
**Total**	70 (44.6)	87 (55.4)	157 (100.0)
**Settlement type (*N* = 157)**			
Formal	19 (12.1)	31 (19.7)	50 (31.8)
Informal	22 (14.0)	46 (29.3)	68 (43.3)
Slum	29 (18.4)	10 (6.4)	39 (24.8)
**Total**	70 (44.6)	87 (55.4)	157 (100.0)
**Education (*N* = 133)** ^b^			
No education	7 (12.3)	10(7.5)	17 (12.8)
Primary education	5 (3.8)	12(9.0)	17 (12.8)
Secondary education	26 (19.5)	32(24.1)	58 (43.6)
Tertiary education	18 (13.5)	23(17.3)	41 (30.8)
**Total**	56 (49.1)	77 (57.9)	133 (100.0)
**Occupation (*N* = 140)** ^b^			
Trading/Business	35 (25.0)	30 (21.4)	65 (46.4)
Artisan/craftwork	17 (12.1)	9 (6.4)	26 (18.6)
Not employed/student/housewife	1 (0.7)	22 (15.7)	23 (16.4)
Professional	6 (4.3)	8 (5.7)	14 (10.0)
Self employed	2 (1.4)	4 (2.9)	6 (4.3)
Others^a^	2 (1.4)	4 (2.9)	6 (4.3)
**Total**	63 (45.0)	77 (55.0)	140 (100.0)

^a^Clergy, security, farmer, retired

^b^Non responses excluded from analysis

### Socio-demographic characteristics of KII participants

Information on the socio-demographic characteristics of the KII participants is presented on Table 2. A total of 40 KII participants were involved in both metropolises and the majority (65.0%) of the KII participants were males. The proportions of participants who were formal healthcare workers, informal healthcare providers and community/opinion leaders were 50.0%, 30.0% and 20.0% respectively. The proportions of KII participants residing in formal settlements, informal settlements and slums across both metropolises were 35.0%, 47.5% and 17.5% respectively. Majority (80.0%) of the participants had tertiary education.

**Table 2:** Socio-demographic characteristics of KII participants [N = 40]

Variable	Ibadan n (%)	Kano n (%)	Total n (%)
**Sex**			
Male	7 (17.5)	19 (47.5)	26 (65.0)
Female	13 (32.5)	1 (2.5)	14 (35.0.)
**Total**	20 (50.0)	20 (50.0)	40 (100.0)
**Category of participants**			
Formal healthcare workers	10 (25.0)	10 (25.0)	20 (50.0)
Informal healthcare providers	6 (15.0%)	6 (15.0%)	12 (30.0%)
Community/Opinion leaders	4 (10.0%)	4 (10.0%)	8 (20.0%)
**Total**	20 (50.0)	20 (50.0)	40 (100.0)
**Settlement type**			
Formal	9 (22.5)	5 (12.5)	14(35.0)
Informal	5 (12.5)	13 (32.5)	18 (45.5)
Slum	6 (15.0)	2 (5.0)	8 (20.0)
**Total**	20 (50.0)	20 (50.0)	40 (100.0)
**Education**			
No education	4 (0.0)	6 (3.3)	10 (25.0)
Primary education	3 (10.0)	1 (0.0)	4 (10.0)
Secondary education	1 (3.3)	1 (3.3)	2 (6.6)
Tertiary education	12 (40.0)	12 (40.7)	24 (80.0)
**Total**	20 (50.0)	20 (50.0)	40 (100.0)

### Opinions about where community members seek care for malaria

#### Seeking care for malaria in health facilities

The expression ‘health facilities’ or ‘hospitals’ were commonly conceptualized and understood by the participants as a generic term for all categories of formal health facilities. Participants’ responses were general and not specifically tailored across the various levels of healthcare although at some point a few participants were specific about the category of health facility referred to during the discussion or interview. One of the dominant viewpoints that cut across several FGDs and KIIs in both Ibadan and Kano metropolises was that many community members prefer to seek “proper” care for suspected malaria infection from government-owned health facilities especially Primary Health Care (PHC) facilities due to proximity, affordability of services being offered as well as availability of qualified health workers in the health facilities. However, there were other FGD sessions where participants opined that the rich and educated in their communities often prefer to seek care for suspected malaria infections in the private hospitals. Additionally, there were instances where a few FGD participants especially from informal settlements in Kano metropolis emphatically disclosed that they prefer to seek care in tertiary health facilities when they experience symptoms suggestive of complicated or severe malaria.

*When we are sick if we have severe malaria, we go to the hospital but when it is just mild sickness we go to chemists (Participant 1, Female, FGD 5, Informal settlement, Ja’en Gidan Gabas, Dorayi, Kano).*

*If we suspect that we have malaria, we go to the health centre for treatment (Participant 3, FGD 6, Slum, Adekile, Agugu ward, Ibadan North East LGA, Ibadan).*

*The place that the community members prefer to go to because of money is the Primary health center, because the private hospitals are a bit expensive but the primary health centres, one can still afford it (Participant 1, FGD 5, Slum, Bibilari/Alugbua Bashorun, Ibadan North LGA, Ibadan).*

*Affordability is what makes them to go to public hospital (KII 12, Drug Peddler, Duniyar Yangaruwa, Gobirawa, Kano).*

*If sick is severe is better to go tertiary hospital not primary healthcare centre (Participant 2, FGD 5, Female, Informal settlement, Ja’en Gidan Gabas, Dorayi, Kano).*

#### Seeking malaria treatment in proprietary patent medicine stores

A common viewpoint expressed by more participants in informal settlements and slums in both metropolises was that most community members often patronise PPMVs or drug sellers as the first step for obtaining malaria care. In the FGDs and KIIs conducted in informal settlements and slum communities, participants admitted that poverty is the main reason their community members prefer to patronize PPMVs for the management of suspected malaria infections. Other factors that were frequently cited especially by KII participants for patronizing PPMVs for treatment of suspected malaria infection were ignorance about ideal treatment sources for suspected malaria infection and non-availability of health services in PHC facilities at night.

*People like buying drugs over the counter when there is no money to seek hospital treatment (Participant 4, Slum, FGD 9, Molete, Ibadan South West, Ibadan).*

*In our community here, patronising drug sellers is the most common because it is how your pocket is that will determine the care you will like for yourself….(KII 14, Traditional Doctor, Slum, Agugu, Ibadan North East, Ibadan).*

*If it is fever we first go to the chemist, it is the first stage. If they went there and they did not feel okay then they will go to the hospital (KII 18, Ward Head, Kastina Road, Fagge LGA, Kano).*

*I first go chemist. Even when I don’t have money I can pay him later (Participant 10, FGD 5, Female, Informal settlement, Ja’en Gidan Gabas, Dorayi, Kano).*

#### Seeking malaria treatment from herbal drug sellers

Some of the KIIs as well as FGD participants, comprising majorly those from informal settlements and slum communities, across both metropolises revealed that some of their community members especially the ‘older people’ prefer to seek treatment for suspected malaria infections from herbal drug sellers or traditional medicine practitioners. The key reasons cited for seeking treatment from herbal drug sellers were poverty or inability to pay hospital bills or buy drugs, cheap cost of herbal drugs, lack of education and the belief that herbal drugs work faster or are more effective. Some FGD participants in Ibadan metropolis particularly noted that they prefer to use herbal drugs because “they believe in the use of herbal drugs”. However, there was an instance where one of the FGD participants in Kano metropolis emphatically declared that traditional medicine does not work for treating malaria infection.

*The elders believe in herbs and that is what they use often (Participant 1, Mother of Under-five, FGD 8, Informal settlement, Eleyele, Olopomewa ward, Ibadan North West, Ibadan).*

*Some residents of this community find it difficult to spend even 10 Naira on sachet water due to poverty not to talk of purchasing drugs, which is why they treat malaria using our own traditional medicines (KII 16, Traditional Doctor, Naira da Kobo, Gobirawa, Kano).*

*Traditional medicine is not working at all (Participant 1, FGD 9, Hauren Gadagi, Zango, Kano).*

### Factors influencing community members’ choice of facility to seek malaria treatment

#### Lack of money for paying health facility/hospital bills

A viewpoint that cuts across the various FGD sessions and KIIs in both metropolises was that lack of money or poverty was a major factor that usually influenced community members’ decision to seek care for suspected malaria infection in a health facility or hospital (the term health facility or hospital is used interchangeably in this context. Hospitals also encompass primary health care facilities). Several FGD participants as well as key informants who were community leaders, interviewed in slum communities, clearly remarked that lack of money often made some of their community members resort to the use of herbal drugs or seeking care from PPMVs. One particular male FGD participant noted that “we do not go to hospital because our economic status is low”.

*Money is influential on whether to go for medical care or not (Participant 8, FGD 1, Male, Formal settlement, Dorayi Babba Layout, Doaryi, Kano).*

*Money is the major thing because if one have money why won’t he go to where one ought to go to (KII 11, Formal settlement, Challenge, Ibadan South West, Ibadan).*

*The issue is sometimes in hospitals the medicine is more expensive than in chemists…And some can't pay bills for treatment (KII 15, PMV, Akija Hotel, Fagge, Kano).*

#### “Proper” treatment

Participants in some FGD sessions in both Ibadan and Kano metropolises opined that “proper” treatment that are usually offered in hospitals (used by many participants to refer to any formal health facilities) especially in government-owned health facilities encourage them to seek treatment for suspected malaria infection in health facilities. Some of the typical expressions that participants used in buttressing their points included: “we go to the hospital to get treatment and we get well”; “at the hospital they will investigate to know what is wrong with you”; “the treatment they offer to us is perfect”; “the treatment they offer is better than that of chemists”.

*Because if you go to the hospital you will get treated but if you stayed at home the problem will get worse, but if you go to hospital you will be well treated and be fine (Participant 4, FGD 4, Female, Formal settlement, Kastina Road, Fagge, Kano).*

*The treatment they offer to us is perfect (Participant 9, FGD 6, Slum, Adekile, Agugu ward, Ibadan North East LGA, Ibadan).*

*What has influence is that when they go, the malaria will be treated and they will be healed (KII 12, Slum, Oluwo/Idi-Itan, Bashorun ward, Ibadan North, Ibadan).*

### Attitude of health workers

An opinion that was commonly shared in some FGDs and KIIs across both metropolises was that unfriendly attitudes of health workers often discourage seeking treatment in the health facilities. Some FGD participants strongly expressed the view that “some health workers in government hospitals especially PHC facilities are arrogant and rude to patients”. A number of participants, majorly FGD participants in slum communities, disclosed that the attitude of health workers towards them is responsible for their not seeking care in the hospital.

*It is the doctor’s negative bad attitudes that make us run from hospital (Participant 8, FGD 3, Slum, Orita-Aperin, Ibadan North East, Ibadan).*

*The attitude of the health workers influences me more because I have been a victim of their bad attitude and that is why I prefer chemists rather than hospital (Participant 9, FGD 3, Male, Informal settlement, Zage, Zango, Kano).*

*It is actually the bills and the attitude of nurses that discourage us (Participant 5, FGD 7, Mothers of Under-five, Formal Settlement, Oluwo-Nla, Bashorun, Ibadan North, Ibadan).*

### Other factors influencing community members’ choice of place malaria treatment is sought

Some other issues mentioned as factors influencing community members’ choice of facility for seeking of care across the various FGD sessions and KIIs in both metropolises were: the provision of free services in the hospital; availability and proximity of health facilities; perception of suspected malaria infection as being severe. Influence of husbands and other significant others, availability of drugs in the health facility, provision of effective drugs and conduct of malaria tests in the health facility were also frequently mentioned as factors which often encourage community members to seek care in the formal health facilities including hospital. Across many FGD sessions and KIIs in both Ibadan and Kano metropolises, cultural beliefs and preference for traditional medicine as well as overcrowding and long waiting time in the health facilities were frequently mentioned as factors discouraging community members from seeking treatment for suspected malaria infection in health facilities/hospitals. Additionally, lack of adequate manpower in the health facility, high cost of transportation to hospital, availability of time and “fear of injection” were mentioned by a few. One particular KII participant, a traditional medicine practitioner, in one of the slum communities in Ibadan metropolis revealed that some of his community members do not seek treatment for suspected malaria infection in the hospital because “they see doing so as a waste of money”.

*Some people look at going to hospital like a waste of money…; but the person that thinks it’s a waste of time feels so because he doesn’t have enough money. He will rather use such money to do business (KII 14, Traditional Doctor, Slum, Agugu, Ibadan North East, Ibadan).*

*A person has to be frequently sick to go to the hospital when it comes to malaria (KII 14, Traditional medicine man, Informal settlement, Tudun Wazurci, Kano).*

### Perception of differences in health seeking for malaria by socio-economic group

#### Differences in the health seeking behaviours of the “rich” and the “poor”

Almost all the key informants and FGDs participants except a few individuals who were residents of a slum community in Ibadan shared the view that there is a difference between the health seeking behaviour of rich and the poor in their communities. The participants commonly opined that the rich in their community could afford to seek care for suspected malaria infection in any health facility of their choice because they have the resources to pay the bills. It was repeatedly mentioned that the rich prefer to seek treatment of suspected malaria infection in private hospitals, while the poor often visit the government-owned health facilities especially PHC facilities that are nearest to them if affordable. It was also alluded to that the rich prefer to seek care from private hospitals because “they do not like their time to be wasted”. Some of the key informants, consisting of PPMVs, noted that while the rich are eager to go to the hospital, the poor often prefer to buy ‘akapo’ which is a combination all sorts of drugs (analgesics, antimalarial, haematinics, anti-inflammatory among others) dispensed in counts for treating suspected malaria infection. Similarly, key informants who were traditional medicine practitioners and herbal drug sellers affirmed that those that usually seek health care for suspected malaria infection from them are majorly the poor.

*Those wealthy in the community go to private hospitals or they go to government secondary and tertiary hospitals (Participant 5, FGD 1, Male, Formal settlement, Dorayi Babba Layout, Doaryi, Kano).*

*Sometimes those who have money do not seek health care in this country, they fly to another country (Participant 6, Female, FGD 6, Informal settlement, Tudun Wazurchi, Kano).*

*A rich man can go from Nigeria to abroad to treat his malaria using millions of Naira. And use that ample opportunity to rest and to take leave from all these Nigeria problems (KII 19, Informal settlement, Idi-Odo, Challenge, Ibadan South West LGA, Ibadan).*

*Rich people are going to private hospitals to seek medical attention but we the poor people are going to government hospitals (Participant 7, FGD 9, Slum, Hauren Gadagi, Zango, Kano).*

*Rich people go to professional doctors for medication, but the majority of the poor come to us… (KII 16, Traditional Doctor, Naira da Kobo, Gobirawa, Kano).*

#### Differences in the health seeking behaviours of the educated and the uneducated

A common viewpoint expressed by nearly all the key informants as well as several FGD participants across both metropolises was that there is a difference between the behaviours of educated people and uneducated people in their communities with regards to seeking care for suspected malaria infection. Many key informant interviewees opined that educated people know the importance of taking good care of themselves, the dangers that can arise from having malaria as well as the value of going to the hospital to do a malaria test. Several FGD participants and key in-depth interviewees including community leaders and formal health workers across both metropolises admitted that educated people prefer to go to private hospitals to seek treatment for suspected malaria infection, while uneducated people prefer to visit government-owned health facilities to obtain treatment for suspected malaria infection. Additionally, some key informants revealed that some uneducated people do not seek proper care for suspected malaria infection because they often associate their ill-health condition with witchcraft or spiritual causation.

*In this community, the educated ones are always eager to visit the hospital than those who are not educated. But there are some uneducated ones that if referred, will go to the hospital. Those who are older and uneducated even if you tell them to go to the health facility they won’t take it seriously (KII 12, Slum, Oluwo/Idi-Itan, Bashorun ward, Ibadan North, Ibadan).*

*…people without formal education are always relating malaria with beliefs like bewitching, or some kind of spiritual issue… (KII 2 Roll-Back Malaria Officer, Fagge LGA, Kano).*

### Opinions about appropriate sources of health care for malaria

#### Visiting the hospital for appropriate malaria treatment

Across both Ibadan and Kano metropolises, many FGD participants shared the opinion that anyone who suffers malaria in the community should go to the health facility/hospital to receive proper treatment. Several participants affirmed that anyone who has suspected malaria infection should not indulge in self-medication but rather consider visiting the health facility/hospital where proper medical diagnosis, testing and treatment can be obtained. The formal healthcare workers interviewed declared that it is only individuals or patients that are malaria positive based on the result of the malaria test that should be prescribed or treated with Artemisinin Combination Therapy (ACT) drug.

*Hospitals are the best to seek treatment (Participant 1, FGD 2, Male, Informal Settlement, Filin Durumi, Gobirawa, Kano).*

*The advice I will give such person is to go to the hospital in order to receive better treatment. He/she should not do self-medication because he/she cannot determine if it is malaria or not, it is until he or she go for test in the hospital…(Participant 5, FGD 1, Formal Settlement, Providence Estate, Olopomewa ward, Ibadan North West LGA, Ibadan).*

*Mostly we, the Volunteer Community Mobilizer (VCM), give advice to the community members that if their children or pregnant women are sick they should quickly go to the hospital and seek medical care (Participant 5, Mother of under-five, FGD 9, Slum, Hauren Gadagi, Zango, Kano).*

#### Use of herbal drugs for treating malaria

Several FGD participants and key informants who were residents of slums and informal settlements in both metropolises, clearly expressed their support for the use of herbal drugs for treating malaria. Some participants particularly affirmed that they believe in the use of herbal drugs and will endorse its use for anyone who has suspected malaria infection. There were instances where some participants claimed that herbal drugs work faster and better than ‘modern drugs’ (orthodox drugs) in treating malaria. A few participants expressed the belief that “herbal drugs help to flush malaria from the body”.

*My own belief is if I use ‘Alabukun’ (an analgesic manufactured by indigenous pharmaceutical company containing 760mg acetylsalicylic acid and 60mg caffeine) mixed togther with “hot” alcoholic herbal drink that is very bitter, I will not feel the malaria again (Participant 3, FGD 2, Informal settlement, Challenge, Ibadan South West, Ibadan).*

*If it is severe someone can first use orthodox tablet to “step down” the severity of the ailment. After that, then use herbs to flush it away. Because tablets does not flush the body, it’s herb that flushes malaria away (Participant 9, FGD 3, Slum, Orita-Aperin, Ibadan North East, Ibadan).*

*As we are seated here, majority don’t believe in hospitals; we have seen people given drips in hospital and at last they still die. So people see hospital as where you will be close to the grave. There is no adequate care in hospitals again, I am always afraid of going to hospital (Participant 8, FGD 3, Slum, Orita-Aperin, Ibadan North East, Ibadan).*

*We first steamed the body with herbs such as “Tazargadi” and hot water and if it is severe we go to the hospital (Participant 2, FGD 5, Female, Informal settlement, Ja’en Gidan Gabas, Dorayi, Kano).*

### Summary of factors influencing care-seeking behaviour for malaria among urban dwellers

**Fig. 1:**
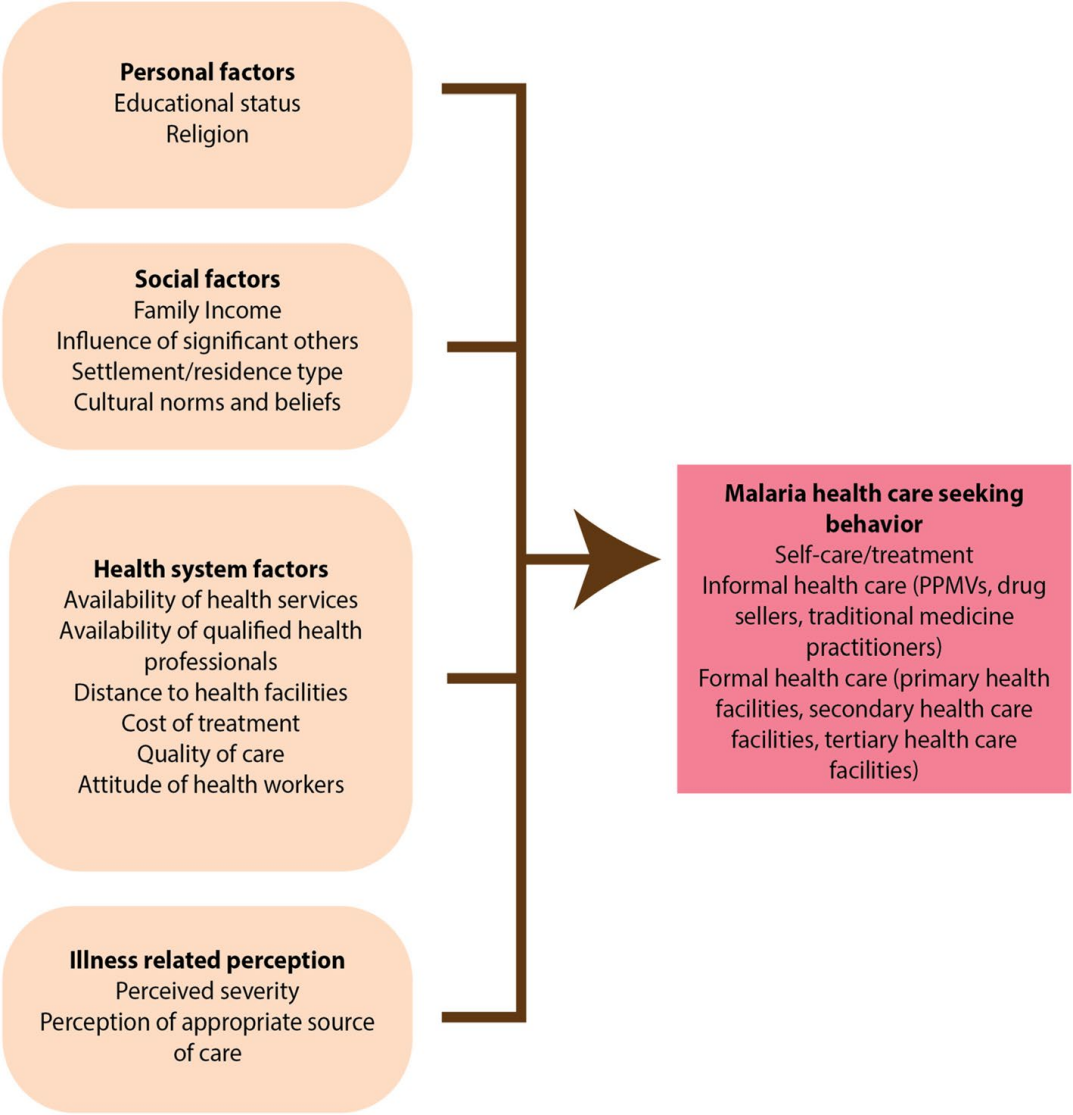
Schema of factors that influence the malaria related care-seeking behaviour of community members

The schematic illustration of the malaria related care-seeking behaviour of community members is shown on Fig. 1. Succinctly put, diverse factors and issues influence malaria care-seeking behaviours of community members. These issues cut across personal factors, social factors, health system factors and illness perceptions, which are themselves, inter-related.

## Discussion

The findings of this study indicate that many community members in urban settlements especially those in informal and slum settlements prefer to seek proper care for suspected malaria infection from government-owned health facilities/hospitals especially PHC facilities than private health facilities due to proximity and affordability of services in government-owned health facilities. Similar pattern of finding has been reported in previous studies [9, 20, 21] conducted in urban areas in Nigeria. In a previous study [20] conducted in Kano metropolis, 74.3% of the participants mentioned that they went to health facilities when they had an episode of fever. This pattern of finding observed in our study is not surprising as PHC facilities are the main channel through which key treatment and prevention interventions for malaria are delivered. The PHC system is the first level of contact for individuals, families and communities with the national health system [22].

This study found that many community members especially in informal settlements and slums often patronize PPMVs or drug peddlers as the first step or point for obtaining care for suspected malaria infection. This is similar to what previous studies [23, 24] reported. It has been noted that going to a PPMV for presumptive treatment of malaria puts community members at high risk of using substandard drugs and non-recommended drugs for malaria as well as use of anti-malarial drugs without undergoing the mandated malaria test [23]. Poverty and ignorance which were cited as the reasons community members patronize PPMVs for care of suspected malaria infections are in tandem with what previous studies have noted [23, 25]. This study reinforces the need to train PPMVs on proper malaria diagnosis, appropriate and treatment of malaria as well as referral to health facilities.

It was noted that some community members, especially the uneducated in slums and informal communities often patronize herbal drug sellers and traditional healers for care of suspected malaria infection. Some previous studies [26–29] reported that many residents of urban slums and informal settlements including young people and even pregnant women rely on herbal medicines or concoctions to prevent malaria or manage suspected malaria infection. The major reasons adduced for the behaviours included affordability of herbal drugs and ‘belief that herbal drugs work faster or more effectively’. Some studies [26, 27] have observed a similar pattern of results regarding factors promoting the use of herbs for malaria care. The belief that the use of herbal drugs is more effective may be misleading and therefore necessitate the need for continuous enlightenment of the populace on appropriate malaria treatment options.

Participants identified a range of factors that prevent seeking of malaria treatment in health facilities to include cost of treatment and distance to health facility. It is not surprising that the cost of treatment is a major barrier to accessing appropriate and quality care for malaria in health facilities as many people in Nigeria are yet to be registered under the national health insurance scheme, and health care costs are majorly out-of-pocket [12]. The results of this study suggest the need for free or subsidized malaria treatment as a veritable strategy towards equitable and early care for malaria**.** When treatment for malaria in the hospital is rendered free or subsidized, location of the health facilities especially the PHC facilities that are meant to be closest to community members also matters; as the cost of transportation to the health facility could constitute a barrier to utilization of service [9]. Community-based case management of malaria using trained community volunteers or means that can be easily accessible by community members has the potential for promoting appropriate and prompt care for suspected malaria infection could be further explored and strengthened as a viable strategy. For instance, in Kano state, the CHIPS (Community Health Influencers, Promoters and Services) programme, a community health worker model for improved community-based management of malaria in hard-to reach-areas can be extended to urban areas to address the challenge of inappropriate care seeking behaviours such as self-treatment and dependence on informal healthcare providers like drug hawkers and traditional doctors.

Cultural beliefs and preference for traditional medicine were also identified as factors affecting seeking of care for suspected malaria infection in health facilities. These findings are in tandem with what previous studies [27, 30] documented as factors that influence community members not to seek care for suspected malaria infection in health facilities. Reliance on the use of herbal drugs for treating suspected malaria infection can make malaria cases to become complicated before visiting a health facility [26]. Major obstacles to the use of herbal drugs are their poor quality control and safety [31]. This points to the need for more research into the safety and efficacy of herbs in the treatment of malaria [32]. Similarly, government needs to increase efforts on phytomedicine and regulations of practices of herbal medicine practitioners as part of the measures towards malaria elimination and control.

Factors such as unfriendly attitude of health workers, long waiting time, lack of adequate manpower in the health facility and non-availability of time for seeking care in a health facility are similar to issues earlier identified in previous studies [8, 30] as factors that are generally affecting community members’ health service utilization in Nigeria. The poor services resulting from long waiting time in health facilities, hostile attitude of some health workers as well as lack of competent health workers have been reported as factors that affect utilization of services in PHC facilities in Nigeria [27, 33]. The finding from this study underscores the need for improvement in healthcare service delivery especially in PHC system, where uncomplicated malaria cases are typically treated. Additionally, some health system related barriers identified like long waiting time in health facilities and hostile attitude of some health workers suggest the use of appropriate health system strengthening actions including periodic capacity building initiatives for health workers, thorough supervision and monitoring, promotion of culture of respect for patients as well as having policies on zero-tolerance for violence.

It was revealed that educated people often prefer to visit health facilities for malaria care. Previous studies [23, 25] have found an association between higher education and timely seeking of treatment for suspected malaria infection in formal healthcare settings. Our study finding showed that many uneducated community members do not care about seeking care for suspected malaria infection from the hospital which is in tandem with what previous studies [23, 34] have found out. It is more likely that the uneducated will have low health literacy and so they may not be aware of appropriate settings to treat their malaria related illness [35]. This finding underscores the need for continued focus on health education on issues relating to malaria to ensure improved and appropriate malaria seeking behaviour.

Our study findings also revealed that some uneducated people do not seek proper care for suspected malaria infection because they often relate their ill-health condition to witchcraft or spiritual causation/causes. Similarly, some previous studies [30, 36] earlier noted that beliefs in the supernatural causes of disease can be barriers to seeking appropriate treatment for suspected malaria infection. Several participants in slum and informal settlements in both metropolises expressed support for the use of herbal drugs as they considered the use of herbal drugs to be the appropriate treatment option for suspected malaria infection, especially the severe form. Some participants maintained the position that herbal drugs are better and more effective than prescription drugs for treating suspected malaria infection. This kind of belief or conviction is not only wrong medically, it is capable of causing serious complications as well as undue exposure to harmful products. The belief expressed by the community members may be due to lack of knowledge about malaria care and control. Community mobilization and public enlightenment are strategies which could be used to address wrong beliefs and misconceptions about issues relating to malaria care.

The interplay of multiple factors encompassing personal factors, social factors, health system related factors and illness related perceptions contribute to the complexity of malaria care seeking behaviour. This finding is consistent with what previous studies [9, 37, 38] conducted in urban communities in Nigeria have reported. Therefore, a combination of approaches and strategies is desirable when designing, implementing and evaluating interventions targeting seeking of proper or appropriate care for suspected malaria infection. Furthermore, this study suggests that investments in social determinants of health and health system pillars should also be considered to improve care seeking for fever.

This study had limitations. It was an exploratory qualitative study and thus the findings are not generalizable. Additionally, while this study involved community stakeholders as well as different categories of health stakeholders in the formal and non-formal health sectors; we did not capture stakeholders at the secondary and tertiary healthcare facilities. They may have provided further insights on issues relating to malaria care seeking behaviours of community members in the metropolises as seen at higher levels of care. We recognize the fact that social desirability bias may be intractable in qualitative research. Some of the strategies that were employed to avoid or limit this in our study included establishing good rapport with participants, assurance of anonymity, use of different probing and indirect questioning techniques, triangulation as well as thorough training of field assistants and regular debriefing sessions.

## Conclusions

The key factors influencing care seeking behaviours for suspected malaria infection include cost of hospital bills, attitude of health workers, waiting time in the hospital and cultural beliefs. The positive behaviour to seek care in health facilities for suspected malaria infection should be encouraged by ensuring access and availability of anti-malaria commodities. In addition, attitudinal change among healthcare workers should be addressed. The issues and antecedent factors relating to differential in care seeking behaviour by settlement types calls for targeted intervention. Multiple public health interventions including public enlightenment campaigns, advocacy, training and policy interventions should be used as appropriate to promote appropriate malaria care seeking for malaria treatment in facilities or among healthcare providers that will ensure appropriate case management of malaria.

## Supplementary Information

Supplementary Material 1. Focus Group Discussion Guide – Caregivers. Key Informant Interview Guide – Formal health workers. Key Informant Interview Guide – Informal health workers. Key Informant Interview Guide – Community leaders.

## Data Availability

The datasets used and/or analysed during the current study is available from the corresponding author on reasonable request.

## Abbreviations

FGD: Focus Group Discussion
KII: Key Informant Interview
MSD: Multi-Stakeholders Dialogue
PHC: Primary Health Care
PPMV: Proprietary Patent Medicine Vendor
RA: Research Assistant
SD: Standard Deviation
WHO: World Health Organization
