# Supplementary material for: Are urban residents seeking appropriate care for malaria? evidence from an exploratory qualitative study in two cities in nigeria
Source: BMC Health Serv Res. 2024 Dec 18;24:1572. doi: 10.1186/s12913-024-12013-9 (PMC11653962; doi:10.1186/s12913-024-12013-9)
Supplement: Supplementary file 1 — Supplementary Material 1. Focus Group Discussion Guide – Caregivers. Key Informant Interview Guide – Formal health workers. Key Informant Interview Guide – Informal health workers. Key Informant Interview Guide – Community leaders. [file 12913_2024_12013_MOESM1_ESM.pdf]

Appendix 1

Field assessment of the burden and determinants of malaria transmission in urban areas – Focus group discussion guide

Who to interview: Groups (8 – 12 persons) of Community members that are homogenous- These include:

1. Male adults
2. Female adults
3. Mothers of under-five children

Introduction

My name is ……………and my colleagues are………….. I am working with the University of Ibadan and Bayero University Kano. We would like your opinion on various issues to enable us understand malaria transmission in urban areas. The information we collect will help the government to plan health services to prevent malaria infections by ensuring you receive suitable interventions. You have been specially invited for this focus group discussion and we thank you for honouring our invitation.

Purpose

The purpose of this focus group discussion is to investigate the following

1. How community members manage suspected malaria infections
2. Where community members seek care for malaria
3. Factors that influence care seeking for malaria in a hospital,
4. How health seeking behaviour and source of care differ by socioeconomic group
5. Local names for malaria medications
6. How showing of pictures or physical packaging of malaria medications can improve participants' recall and answers about usage of malaria medication
7. Factors that influence decisions about what malaria medicine to use
8. Common methods that people use to protect themselves from themselves from malaria
9. Understand facilitators and barriers to participating in a community-based disease reporting programme for malaria

The information learned in this focus group will be used to guide the development of other components of our study including surveys and longitudinal component that we are planning to conduct.

Procedure

As part of this study, you will be placed in a group of 8 – 12 individuals. The focus group discussion which will last for 45 – 90 minutes will entail asking you some open-ended questions. In addition to the discussion questions, we will be asking for some of your socio-demographic information. In the course of this discussion, your views will be respected and will not be used against you in any way. This discussion will be taped, so please speak up and speak clearly. We ask for your consent to record the discussion so that we will not miss out anything from the information you will be providing to us through the discussion. Please do share your views without mentioning people's names. We want the discussion to be anonymous and to be confidential as possible. You can choose whether or not to participate in the focus group discussion, and you may stop at any time during the course of the study. There is no right or wrong view, so feel free to express yourself. Out of respect, please refrain from interrupting others. However, feel free to be honest even when your responses counter those of other group members. Remember your participation in this discussion is voluntary. Your decision not to be involved or drop out at any point will not attract any penalty.

Benefits

Your participation in this study may not provide any personal benefit to you. However, should you decide to participate in this study, you will be doing society a great service because the findings of this study will be useful in the design of interventions and programmes for the control and prevention of malaria.

Risks

There are no known or anticipated risks associated with participation in this study beyond those experienced during an average conversation. If a question, or the discussion, makes you uncomfortable, you can choose not to answer.

Confidentiality

Should you choose to participate in the study you will be asked to respect the privacy of other focus group discussion members by not disclosing any content discussed during the study. The information you share will be kept confidential. Identifying information will be removed from the transcripts. The transcripts and other electronic data will be retained for a maximum of 5 years, after which they will be destroyed. Data will be stored in an encrypted folder on protected laptop. Only the research team will have access to study data. No identifying information will be used in any presentations or publications based on this research. Although we will ask all participants in your focus group to maintain confidentiality, we cannot guarantee that they will do so.

Contact

If you have any questions or concerns regarding this study, please contact:
Professor IkeOluwapo Ajayi
Email: ikeyajayi2003@yahoo.com, Tel: 08023268431
IMARAT, College of Medicine, University of Ibadan

Thank you for choosing to participate in the study. Kindly show by using any of the following two boxes, that your participation in this study was voluntary.

☐ I will participate

☐ I will not participate

S/N	Main questions	Follow up questions or hints
	**Introduction and general questions**	
1	a) What can you say about the living conditions of community members?	• Settlements or housing conditions in the community<br>○ (**Probe for**, if not mentioned, presence of formal settlements, informal settlements, slums)<br>○ (Probe for their opinions about features of formal settlements, informal settlements, slums)<br>• Financial condition or status of community members<br>○ (**Probe for**, opinions about categorization of community members based on economic or financial status)<br>• Health facilities in the communities<br>○ **Probe for**, availability of the health facilities in the communities<br>○ **Probe for**, types of health facilities most preferred by community members (private hospital, primary health facilities, secondary health facilities, tertiary health facilities)<br>○ **Probe for**, characteristics of preferred health facilities
	b) Where does the community members generally prefer to go for health care?	**Probe** for the following (if not mentioned):<br>• Health facilities (private hospitals, primary health facility, secondary health facilities, tertiary health facilities). Probe for names of the preferred facilities<br>• Patent medicine stores<br>• Traditional healing home<br>• Drug peddlers)
	c) In this community if someone is pregnant, where would they typically go for antenatal care?	**Probe** for the following (if not mentioned):<br>• Health facilities (private hospitals, primary health facility, secondary health facilities, tertiary health facilities). Probe for names of the preferred facilities<br>• Traditional Birth Attendants<br>• Traditional doctors<br>• Faith-based maternity homes<br>• No where/prefer to give birth at home<br>• Opinions on why some pregnant women prefer not to attend antenatal care in the hospitals
	d) What are the common diseases in this community	• Which diseases are most common among under-five children?<br>• Which diseases are most common among adult population?<br>• Which diseases are the most severe diseases?<br>• Which diseases are associated with mosquito bites in the community?
	**Basic understanding of malaria**	

2	Now let us discuss specifically on some basic issues relating to malaria.<br><br>a) How common is malaria in this community?	 <br><br>What categories of persons are most affected by malaria?<br>Probe for:<br>○ Under-five children<br>○ Adolescents<br>○ Adults<br>○ Aged people<br>○ Pregnant women<br>○ People with sickle cell anemia e.t.c.
	b) How do community members usually get to know that they have malaria?	**Probe** for the following (if not mentioned):<br>○ Through observation of signs and symptoms that are suggestive of malaria<br>○ Clinical examination<br>○ Lab examination
	c) In your opinion, what are the signs and symptoms of malaria	
	d) Please share with us what you think is the cause of malaria?	**Probe** for the following:<br>○ Mosquito bite<br>○ Exposure to hot sunshine<br>○ Poor hygiene<br>○ Hunger<br>○ Spiritual reasons e.t.c
	**Practices relating to management of malaria in the community**	
3	a) How do adult members in community manage suspected malaria infections?	**Probe** for the following (if not mentioned):<br>○ Home-based care<br>○ Self-medication<br>○ Use of herbs<br>○ Use of drugs or medicines with prescription
	b) Tell us about how suspected malaria infections in under-five children are being managed in the community	**Probe** for the following (if not mentioned):<br>○ Home-based care<br>○ Self-medication<br>○ Use of herbs<br>○ Use of drugs or medicines with prescription
	c) Tell us about how suspected malaria infections in adolescents are being managed in the community	**Probe for the following (if not mentioned):**<br>○ Home-based care<br>○ Self-medication<br>○ Use of herbs<br>○ Use of drugs or medicines with prescription

	d) If you think someone in your community has malaria, what kind of treatment should they follow?	**Probe** for the following (if not mentioned):<br>○ Home-based care<br>○ Self-medication<br>○ Use of herbs<br>○ Use of drugs or medicines with prescription
	e) Kindly share your experience of how you managed your last or most recent malaria episode	**Probe for**,<br>• The malaria medication(s) used the last time participants had malaria infection
	**Malaria related health-seeking behaviours of community members**	
4	Where do community members seek care for malaria?	**Probe** for the following (if not mentioned)<br>○ Traditional healing homes<br>○ Patient Medicine Vendors stores<br>○ Drug hawkers/peddlers<br>○ Pharmacy stores<br>○ Herbal drug stores/kiosks<br>○ Hospitals (Private hospital, government hospital)<br><br>**Probe for** where community members seek care for malaria the most and reasons<br><br>**Probe for** where participants treated their own last malaria episodes
5	What can you say about what influences community members to seek malaria treatment in a hospital?	**Probe** for the following<br>• Money for paying bills for treatment<br>• Money for transportation to hospital<br>• Distance of hospital<br>• Access to hospital<br>• Availability of hospital<br>• Availability of medications<br>• Waiting time in the hospital/health facility<br>• Attitude of health workers<br>• Preference for traditional medicine<br>• Preference for a particular type of hospital (Private hospital, primary health centre, secondary health care facility)<br>• Cultural norms and beliefs about seeking care in hospital<br>• Cultural norms and beliefs malaria<br>• Perceived seriousness of malaria<br>• Perceived threat of malaria<br>• Frequency of malaria episode
6	What can you say about what influences community members to seek malaria treatment in a particular hospital/health facility?	Probe for the following<br>• Attitude of the health workers<br>• Reputation of the provider<br>• Previous experience with provider<br>• Gender of the provider<br>• Distance to the health facility

		• Quality of services<br>• Ambience of health facility<br>• Waiting times<br>• Availability of medication<br>• Cost of services
7	Let us briefly discuss how health seeking behaviour and source of care differ by socioeconomic group?	**Probe** for –where each of the following categories of people usually seek malaria care?<br>• Rich people in the community<br>• Poor people in the community<br>• People with formal education<br>• People without formal education<br><br>(**For each of the categories, probe for the common source of malaria treatment e.g** - government institutions, private clinics, pharmacy, chemists, drug peddlers/hawkers, traditional healing homes, herbal drug stores/kiosks)
	**Malaria medications being used and their local names**	
8	a) What can you say about the malaria medications that community members use?	**Probe** for<br>• Please tell us about malaria medications that are commonly used among adult population in the community<br>• Tell us about the malaria medications that are commonly used among children in the community<br>• What are your views about the cost of the malaria medications
	b) What are the local names for the malaria medications that community members use?	
	**Showing of pictures or physical packaging of malaria medications to improve recall and answers about usage of malaria medication**	
9	a) What are your opinions about the showing of pictures or physical packaging of malaria medications with the intention of improving recall and answers about usage of malaria medication?	**Probe** for<br>• Why they think showing of pictures or physical packaging of malaria medications can possibly improve recall and answers about usage of malaria medication?
	b) In your opinion what other ways or means do you think can help community members to improve recall and answer about usage of malaria medications?	
	**Factors influencing use of malaria medications and treating malaria at a hospital**	
10	a) Please share with us the things that do influence community members decisions about choice of malaria medications	**Probe** for<br>• **Individual factors that influence the decisions about what malaria medicine to use – e.g** Age, sex (male or female), marital status, economic status, being pregnant, medical history, perceived

		seriousness of malaria, perceived threat of malaria, preference for particular drug type<br>• **Drug related factors that influence the decisions about what malaria medicine to use –** e.g Availability of drug in hospital or drug stores, number of days expected to use drugs, number of capsules/dosages, taste of drug, side effects of drugs, drug resistance, proliferation of fake malaria medicines, giving of genuine malaria medications<br>• **Social-cultural factors that influence the decisions about what malaria medicine to use –** e.g support from partners, support family members, support from friends/peers, media influence and drug adverts, cultural norms and values<br>• **Health system related factors that influence the decisions about what malaria medicine to use-** e.g availability of drugs in hospital/pharmacy, prescription of drug, advice from health workers<br>• **Policy related factors e.g** –governments and regulatory bodies recommendation relating to antimalarial drugs that should be first line of treatment<br>• **Disease pattern related factors e.g** –uncomplicated vs severe malaria<br>• **Economic related factors** e.g – cost of buying malaria medications, affordability of preferred malaria medications
	b) What are the foremost things or situations that do influence your own decisions about the use of malaria medications?	**Probe for:**<br>• Cost of treatment in private hospitals<br>• Cost of treatment in primary health facilities<br>• Cost of treatment in secondary health facilities<br>• Cost of treatment in patent medicine store<br>• Cost of treatment using traditional/herbal medicines
	c)What can you say about the cost of treating malaria in the hospital?	• How affordable do you think the cost of treating malaria is for community members in the health facility?
	**Common methods that community members use to protect themselves from malaria**	
11	a) Please tell us about the common methods that people use to protect themselves from malaria	**Probe** for (if not mentioned):<br>• Use of insecticide sprays<br>• Use of insecticides treated bed nets<br>• Use of mosquito replants<br>• Use of coils<br>• Use of window and door screens<br>• Wearing of long-sleeved clothing and long pants<br>• Malaria prophylaxis

	**b)** Which method is most commonly used by people to protect themselves from malaria?	**Probe** for<br>• Reasons for being the most commonly used
	**c)** Which methods do community members usually use to protect under-five children from malaria?	
	**Participation in community-based malaria programme**	
12	a) We are planning to put in place a free community-based programme whereby community members will be asked to consistently report malaria cases or symptoms through their cell phones. Our goal is to understand the transmission of malaria in the community to inform where interventions go. What is your opinion about how community members will perceive the programme?	
	b) What can facilitate the participation of community members in the programme?	
	c) Supposed we ask community members to be reporting malaria cases or symptoms via text message as part of our community-based malaria programme, what is your opinion about it?	**Probe** for:<br>• How easy it will be for community members to participate<br>• How willing community members will be<br>• The barriers that may be associated with it<br>• What can encourage community members to be reporting consistently malaria cases or symptoms via text message<br>• Participants' willingness to participate to be involved
	d) What is your opinion about asking community members to be reporting malaria cases or symptoms via a mobile application as part of our community-based malaria programme?	**Probe** for:<br>• How easy it will be for community members to participate<br>• How willing community members will be<br>• The barriers that may be associated with it<br>• What can encourage community members to be reporting consistently malaria cases or symptoms via a mobile application<br>• Participants' willingness to participate to be involved
	e) If we want community members to be reporting malaria cases or symptoms consistently what would be the preferred way or means for this?	**Probe** for (if not mentioned):<br>• Face-to-face with a project volunteer<br>• Through community representative<br>• Through SMS<br>• Through WhatsApp

		• Through mobile application<br>• Through hotline<br>• Through community meeting etc.
	**Conclusion and other relevant information**	
13	a) Please tell us about on-going or existing community-based malaria interventions in this community	
	b) What suggestions do you have about how malaria can be controlled in this community?	
	c) What other suggestions do you have that can help us with the community-based malaria programme that are planning?	

Socio-demographic information

Ward and LGA…………………………… Name of Community/Area…………………

Age in years (at last birthday) ……….. Sex……………………

Highest level of Education……………… Primary Occupation………………………..

Type of community/settlement …………………………………

Appendix 2

Field assessment of the burden and determinants of malaria transmission in urban areas – key informant interview guide for formal health care workers

Who to interview: Formal healthcare providers- These include:

1. Heads of Primary Health Care (PHC) Centres/facilities
2. PHC coordinators /Medical officer of Health at LGA level
3. Roll-back malaria focal persons at LGA level
4. Pharmacists and doctors working in private and public health facilities
5. Malaria programme officers at State level

Introduction

My name is ……………and my colleagues are…………… I am working with the University of Ibadan and Bayero University Kano. We would like your opinion on various issues to enable us understand malaria transmission in urban areas. The information we collect will help the government to plan health services to prevent malaria infections by ensuring you receive suitable interventions. You have been specially invited for this key informant interview and we thank you for honouring our invitation.

Purpose

The purpose of this key informant interview is to investigate the following

1. How community members manage suspected malaria infections
2. Where community members seek care for malaria
3. Factors that influence care seeking for malaria in a hospital,
4. How health seeking behaviour and source of care differ by socioeconomic group
5. Local names for malaria medications
6. How showing of pictures or physical packaging of malaria medications can improve participants' recall and answers about usage of malaria medication
7. Factors that influence decisions about what malaria medicine to use
8. Common methods that people use to protect themselves from themselves from malaria
9. Understand facilitators and barriers to participating in a community-based disease reporting programme for malaria

The information learned in this key informant interview will be used to guide the development of other components of our study including surveys and longitudinal component that we are planning to conduct.

Procedure

The key informant interview, which will last for 40 – 60 minutes, will entail asking you some open-ended questions. In addition to the interview questions, we will be asking for some of your socio-demographic information. In the course of this interview, your views will be respected and will not be used against you in any way. This interview will be taped, so please speak up and speak clearly. We ask for your consent to record the interview so that we will not miss out anything from the information you will be providing to us through the interview. Please do share your views without mentioning people's names. We want the interview to be anonymous and to be confidential as possible. You can choose whether or not to participate in the interview, and you may stop at any time during the course of the study. There is no right or wrong view, so feel free to express yourself. Please note that your participation in this interview is voluntary. Your decision not to be involved or drop out at any point will not attract any penalty.

Benefits

Your participation in this study may not provide any personal benefit to you. However, should you decide to participate in this study, you will be doing society a great service because the findings of this study will be useful in the design of interventions and programmes for the control and prevention of malaria.

Risks

There are no known or anticipated risks associated with participation in this study beyond those experienced during an average conversation. If a question, or the discussion, makes you uncomfortable, you can choose not to answer.

Confidentiality

The information you share will be kept confidential. Identifying information will be removed from the transcripts. The transcripts and other electronic data will be retained for a maximum of 5 years, after which they will be destroyed. Data will be stored in an encrypted folder on protected laptop. Only the research team will have access to study data. No identifying information will be used in any presentations or publications based on this research.

Contact

If you have any questions or concerns regarding this study, please contact:
Professor IkeOluwapo Ajayi
Email: ikeyajayi2003@yahoo.com, Tel: 08023268431
Director, Institute for Advanced Medical Research and Training (IAMRAT),
College of Medicine, University of Ibadan

Thank you for choosing to participate in the study. Kindly show by using any of the following 2 boxes, that your participation in this study was voluntary.

I will participate	I will not participate
☐	☐

S/N	**Main questions**	**Follow up questions or hints**

	**Introduction and general questions**	
1	a) Please tell us about the health facilities that are available in this community	**Probe for:**<br>• Primary health centres<br>• Secondary health facilities<br>• tertiary health facilities<br>• Private hospitals
	b)Where does the community members generally prefer to go for health care?	**Probe** -(if not mentioned):<br>• Health facilities (private hospitals, primary health facility, secondary health facilities, tertiary health facilities).<br>○ Probe for, opinion about the types of health facilities most preferred by community members<br>• Patent medicine stores<br>• Traditional healing home<br>• Drug peddlers)
	c) In this community if someone is pregnant, where would they typically go for antenatal care?	**Probe** for the following (if not mentioned):<br>• Health facilities (private hospitals, primary health facility, secondary health facilities, tertiary health facilities). Probe for names of the preferred facilities<br>• Traditional Birth Attendants<br>• Traditional doctors<br>• Faith-based maternity homes<br>• No where/prefer to give birth at home<br>• Opinions on why some pregnant women prefer not to attend antenatal care in the hospitals
	d) What are the common diseases in this community	• Which diseases are most common among under-five children?<br>• Which diseases are most common among adult population?<br>• Which diseases are the most severe diseases?
	**Basic understanding of malaria**	
2	Now let us discuss specifically on some basic issues relating to malaria.<br><br>a) How common is malaria in this community?	What categories of persons are most affected by malaria?<br><br>Probe for:<br>○ Under-five children<br>○ Aged people<br>○ Pregnant women<br>○ People with sickle cell anemia e.t.c
	b) How do community members usually get to know that they have malaria?	**Probe** for the following (if not mentioned):

		○ Through observation of signs and symptoms that are suggestive of malaria<br>○ Clinical examination<br>○ Lab examination
	c) In your opinion, what are the signs and symptoms of malaria	
	**Practices relating to management of malaria in the community**	
3	a) How do adult members in community manage suspected malaria infections?	**Probe** for the following (if not mentioned):<br>○ Home-based care<br>○ Self-medication<br>○ Use of herbs<br>○ Use of drugs or medicines with prescription
	b) Tell us about how suspected malaria infections of under-five children are being managed in the community?	**Probe** for the following (if not mentioned):<br>○ Home-based care<br>○ Self-medication<br>○ Use of herbs<br>○ Use of drugs or medicines with prescription
	c) Tell us about how suspected malaria infections in adolescents are being managed in the community	**Probe for the following (if not mentioned):**<br>○ Home-based care<br>○ Self-medication<br>○ Use of herbs<br>○ Use of drugs or medicines with prescription
	d) Kindly describe to us how malaria infection is being managed in the health facility	
	**Malaria related health-seeking behaviours of community members**	
4	Where do community members seek care for malaria?	**Probe** for the following (if not mentioned)<br>○ Traditional healing homes<br>○ Patient Medicine Vendors stores<br>○ Drug hawkers/peddlers<br>○ Pharmacy stores<br>○ Herbal drug stores/kiosks<br>○ Hospitals (Private hospital, government hospital)<br>**Probe for** where community members seek care for malaria the most and reasons
5	What do you think usually influence community members to seek malaria in a hospital?	**Probe** for the following<br>• Money for paying bills for treatment<br>• Money for transportation to hospital<br>• Distance of hospital<br>• Access to hospital<br>• Availability of hospital<br>• Availability of medications

		- Waiting time in the hospital/health facility - Attitude of health workers - Preference for traditional medicine - Preference for a particular type of hospital (Private hospital, primary health centre, secondary health care facility) - Cultural norms and beliefs about seeking care in hospital - Cultural norms and beliefs malaria - Perceived seriousness of malaria - Perceived threat of malaria - Frequency of malaria episode
6	Let us briefly discuss how health seeking behaviour and source of care differ by socioeconomic group?	**Probe** for –where each of the following categories of people usually seek malaria care? - Rich people in the community - Poor people in the community - People with formal education - People without formal education (**For each of the categories, probe for the common source of malaria treatment e.g** - government institutions, private clinics, pharmacy, chemists, drug peddlers/hawkers, traditional healing homes, herbal drug stores/kiosks)
	**Malaria medications being used and their local names**	
7	c) What can you say about the malaria medications that community members use?	- Please tell us about malaria medications that are commonly used among adult population in the community. **Probe for**, the local names for the malaria medications being used by adults in the community - Tell us about the malaria medications that are commonly used among children in the community. **Probe for**, the local names for the malaria medications being used by children in the community - What are your views about the cost of the malaria medications? - How affordable do you think the cost of malaria medications is for community members?
	d) Please think about it if you were sick, which malaria medications would you take	**Probe for**, - The local names for the malaria medications - The malaria medication(s) used the last time participant had malaria infection
	**Showing of pictures or physical packaging of malaria medications to improve recall and answers about usage of malaria medication**	

8	a)What are your opinions about the showing of pictures or physical packaging of malaria medications with the intention of improving recall and answers about usage of malaria medication?	**Probe** for:<br>• Why showing of pictures or physical packaging of malaria medications can possibly improve recall and answers about usage of malaria medication?
	b) In your opinion what other ways or means do you think can help community members to improve recall and answer about usage of malaria medications?	
	**Factors influencing use of malaria medications and treating malaria at a hospital**	
9	a) Please share with us the things that you think do influence community members decisions about choice of malaria medications	**Probe** for<br>• **Individual factors that influence the decisions about what malaria medicine to use – e.g** Age, sex (male or female), marital status, economic status, being pregnant, medical history, perceived seriousness of malaria, perceived threat of malaria, preference for particular drug type<br>• **Drug related factors that influence the decisions about what malaria medicine to use –** e.g Availability of drug in hospital or drug stores, number of days expected to use drugs, number of capsules/dosages, taste of drug, side effects of drugs, drug resistance, proliferation of fake malaria medicines, recognizing genuine malaria medications<br>• **Social-cultural factors that influence the decisions about what malaria medicine to use –** e.g support from partners, support family members, support from friends/peers, media influence and drug adverts, cultural norms and values<br>• **Health system related factors that influence the decisions about what malaria medicine to use-** e.g availability of drugs in hospital/pharmacy, prescription of drug, advice from health workers<br>• **Policy related factors e.g** –governments and regulatory bodies recommendation relating to antimalarial drugs that should be first line of treatment<br>• **Disease pattern related factors e.g** – uncomplicated vs severe malaria<br>• **Economic related factors** e.g – cost of buying malaria medications, affordability of preferred malaria medications

	b) What is your view about the cost of treating malaria in the hospital/health facility?	• How affordable do you think the cost of treating malaria is for community members in the health facility or hospitals?
	**Common methods that community members use to protect themselves from malaria**	
10	a) Please tell us about the common methods that people use to protect themselves from malaria in this community	**Probe** for (if not mentioned):<br>• Use of insecticide sprays<br>• Use of insecticides treated bed nets<br>• Use of mosquito replants<br>• Use of coils<br>• Use of window and door screens<br>• Wearing of long-sleeved clothing and long pants<br>• Malaria prophylaxis
	**b)** Which method is most commonly used by people to protect themselves from malaria?	**Probe** for<br>• Reasons for being the most commonly used
	**c)** Which methods do community members usually use to protect under-five children from malaria?	
	**Participation in community-based malaria programme**	
11	a) We are planning to put in place a free community-based programme whereby community members will be asked to consistently report malaria cases or symptoms through their cell phones. Our goal is to understand the transmission of malaria in the community to inform where interventions go. What is your opinion about how community members will perceive the programme	
	b) What can facilitate the participation of community members in the programme	
	c) Supposed we ask community members to be reporting malaria cases or symptoms via text message as part of our community-based malaria programme, what is your opinion about it?	**Probe** for:<br>• How easy do you think it will be for community members to participate?<br>• How willing do you think community members will be?<br>• What barriers do you think may be associated with it?<br>• What do you think we can encourage community members to be reporting consistently malaria cases or symptoms via text message?

	d) What is your opinion about asking community members to be reporting malaria cases or symptoms via a mobile application as part of our community-based malaria programme?	**Probe** for:<br>• How easy do you think it will be for community members to participate?<br>• How willing do you think community members will be?<br>• What barriers do you think may be associated with it?<br>• What do you think we can encourage community members to be reporting consistently malaria cases or symptoms via a mobile application?
	e) If we want community members to be reporting malaria cases or symptoms consistently what do you think would be the preferred way or means for this?	**Probe** for (if not mentioned):<br>• Face-to-face with a project volunteer<br>• Through community representative<br>• Through SMS<br>• Through WhatsApp<br>• Through mobile application<br>• Through hotline<br>• Through community meeting etc.
	**Conclusion and other relevant information**	
12	a) Please tell us about on-going or existing community-based malaria interventions in this community	
	b) What suggestions do you have about how malaria can be controlled in this community?	
	c) What other suggestions do you have that can help us with the community-based malaria programme that are planning?	

Socio-demographic information

Ward and LGA ……………………………… Sex: ……………………

Age in years (at last birthday) ………… Highest level of Education………………

Designation …………………… Position ……………………

Number of years spent in present position ……………………

Number of years spent in health facility……………………….

Type of settlement ……………………………………………......

Name of Community/Area………………………………………

Appendix 3

Field assessment of the burden and determinants of malaria transmission in urban areas – key informant interview guide for informal health care workers

Who to interview: Informal healthcare providers- These include:

4. Patient Medicine Vendors
5. Drug peddlers/hawkers
6. Herbal drug sellers
7. Traditional doctors

Introduction

My name is …………..and my colleagues are………….. I am working with University of Ibadan, Bayero University Kano, Osun State University and Northwestern University; Chicago. We would like your opinion on various issues to enable us understand malaria transmission in urban areas. The information we collect will help the government to plan health services to prevent malaria infections by ensuring you receive suitable interventions. You have been specially invited for this key informant interview and we thank you for honouring our invitation.

Purpose

The purpose of this key informant interview is to investigate the following

1. How community members manage suspected malaria infections
2. Where community members seek care for malaria
3. Factors that influence care seeking for malaria in a hospital,
4. How health seeking behaviour and source of care differ by socioeconomic group
5. Local names for malaria medications
6. How showing of pictures or physical packaging of malaria medications can improve participants' recall and answers about usage of malaria medication
7. Factors that influence decisions about what malaria medicine to use
8. Common methods that people use to protect themselves from themselves from malaria
9. Understand facilitators and barriers to participating in a community-based disease reporting programme for malaria

The information learned in this key informant interview will be used to guide the development of other components of our study including surveys and longitudinal component that we are planning to conduct.

Procedure

The key informant interview, which will last for 40 – 60 minutes, will entail asking you some open-ended questions. In addition to the interview questions, we will be asking for some of your socio-demographic information. In the course of this interview, your views will be respected and will not be used against you in any way. This interview will be taped, so please speak up and speak clearly. We ask for your consent to record the interview so that we will not miss out anything from the information you will be providing to us through the interview. Please do share your views without mentioning people's names. We want the interview to be anonymous and to be confidential as possible. You can choose whether or not to participate in the interview, and you may stop at any time during the course of the study. There is no right or wrong view, so feel free to express yourself. Please note that your participation in this interview is voluntary. Your decision not to be involved or drop out at any point will not attract any penalty.

Benefits

Your participation in this study may not provide any personal benefit to you. However, should you decide to participate in this study, you will be doing society a great service because the findings of this study will be useful in the design of interventions and programmes for the control and prevention of malaria.

Risks

There are no known or anticipated risks associated with participation in this study beyond those experienced during an average conversation. If a question, or the discussion, makes you uncomfortable, you can choose not to answer.

Confidentiality

The information you share will be kept confidential. Identifying information will be removed from the transcripts. The transcripts and other electronic data will be retained for a maximum of 5 years, after which they will be destroyed. Data will be stored in an encrypted folder on protected laptop. Only the research team will have access to study data. No identifying information will be used in any presentations or publications based on this research.

Contact

If you have any questions or concerns regarding this study, please contact:

Professor IkeOluwapo Ajayi
Email: ikeyajayi2003@yahoo.com, Tel: 08023268431
Director, Institute for Advanced Medical Research and Training (IMARAT),
College of Medicine, University of Ibadan

Thank you for choosing to participate in the study. Kindly show by using any of the following 2 boxes, that your participation in this study was voluntary.

I will participate	I will not participate
☐	☐

S/N	Main questions	Follow up questions or hints
	**Introduction and general questions**	
11	b) What can you say about the living conditions of community members?	• Financial condition or status of community members<br>○ (**Probe for**, opinions about categorization of community members based on economic or financial status)<br>• Health facilities available in the communities<br>○ **Probe for**, types of health facilities (private hospital, primary health facilities, secondary health facilities, tertiary health facilities)<br>○ **Probe for**, the most preferred by community members (private hospital, primary health facilities, secondary health facilities, tertiary health facilities)
	b) Where does the community members generally prefer to go for health care?	**Probe** -(if not mentioned):<br>• Health facilities (private hospitals, primary health facility, secondary health facilities, tertiary health facilities).<br>• Patent medicine stores<br>• Traditional healing home<br>• Drug peddlers e.t.c
	c) In this community if someone is pregnant, where would they typically go for antenatal care?	**Probe** for the following (if not mentioned):<br>• Health facilities (private hospitals, primary health facility, secondary health facilities, tertiary health facilities).<br>• Traditional Birth Attendants<br>• Traditional doctors<br>• Faith-based maternity homes<br>• No where/prefer to give birth at home<br>○ Opinions on why some pregnant women prefer not to attend antenatal care in the hospitals
	d) What are the common diseases in this community	• Which diseases are most common among under-five children?<br>• Which diseases are most common among adult population?<br>• Which diseases are the most severe diseases?
	**Basic understanding of malaria**	
2	Now let us discuss specifically on some basic issues relating to malaria.<br><br>a) How common is malaria in this community?	What categories of persons are most affected by malaria?<br>Probe for:<br>○ Under-five children<br>○ Aged people<br>○ Pregnant women<br>○ People with sickle cell anemia e.t.c

	b) How do community members usually get to know that they have malaria?	**Probe** for the following (if not mentioned):<br>○ Through observation of signs and symptoms that are suggestive of malaria<br>○ Clinical examination<br>○ Lab examination
	c) In your opinion, what are the signs and symptoms of malaria	
	**Practices relating to management of malaria in the community**	
3	a) How do adult members in community manage suspected malaria infections?	**Probe** for the following (if not mentioned):<br>○ Home-based care<br>○ Self-medication<br>○ Use of herbs<br>○ Use of drugs or medicines with prescription
	b) Tell us about how suspected malaria infections of under-five children are being managed in the community?	**Probe** for the following (if not mentioned):<br>○ Home-based care<br>○ Self-medication<br>○ Use of herbs<br>○ Use of drugs or medicines with prescription
	c) Tell us about how suspected malaria infections in adolescents are being managed in the community	**Probe for the following (if not mentioned):**<br>○ Home-based care<br>○ Self-medication<br>○ Use of herbs<br>○ Use of drugs or medicines with prescription
	**(For herbal drug sellers and traditional doctors)**<br>d i) Kindly describe to us how malaria infection should be managed or treated using herbal malaria drugs?	**Probe for:**<br>• Malaria infections involving adults<br>• Malaria infections involving under-five children<br>• Malaria infection involving pregnant women<br>• Complicated malaria infections<br>• Individuals having frequent malaria episodes
	**(For Patient Medicine Vendors and Drug peddlers/hawkers involved in selling over-the-counter malaria medicines)**<br>d ii) Kindly describe to us how malaria infection should be managed or treated using malaria medicines (orthodox medicine)?	**Probe for:**<br>• Malaria infections involving adults<br>• Malaria infections involving under-five children<br>• Malaria infection involving pregnant women<br>• Complicated malaria infections<br>• Individuals having frequent malaria episodes
	**Malaria related health-seeking behaviours of community members**	

<table>
<tr><td>4</td><td>Where do community members seek care for malaria?</td><td>Probe for the following (if not mentioned)

o Traditional healing homes
o Patient Medicine Vendors stores
o Drug hawkers/peddlers
o Pharmacy stores
o Herbal drug stores/kiosks
o Hospitals (Private hospital, government hospital)

Probe for where community members seek care for malaria the most and reasons</td></tr>
<tr><td rowspan="3">5</td><td>a) What do you think usually influence community members to seek malaria care in a hospital?</td><td>Probe for the following

• Money for paying bills for treatment
• Money for transportation to hospital
• Distance of hospital
• Access to hospital
• Availability of hospital
• Attitude of health workers
• Preference for traditional medicine
• Preference for a particular type of hospital (Private hospital, primary health centre, secondary health care facility)
• Cultural norms and beliefs about seeking care in hospital
• Cultural norms and beliefs malaria
• Perceived seriousness of malaria
• Perceived threat of malaria
• Frequency of malaria episode</td></tr>
<tr><td>b) What do you think usually influence community members to seek malaria treatment in Patient Medicine Vendors stores or from drug peddlers?

(For Patient Medicine Vendors and Drug peddlers/hawkers involved in selling over-the-counter malaria medicines)</td><td>Probe for the following

• Money for paying bills for treatment
• Money for transportation to hospital
• Distance of hospital
• Access to hospital
• Availability of hospital
• Attitude of health workers
• Cultural norms and beliefs about seeking care in hospital
• Cultural norms and beliefs malaria
• Perceived seriousness of malaria
• Perceived threat of malaria
• Frequency of malaria episode</td></tr>
<tr><td>c) What do you think usually influence community members to seek malaria in a hospital?</td><td>Probe for the following

• Money for paying bills for treatment
• Money for transportation to hospital
• Distance of hospital
• Access to hospital
• Availability of hospital
• Attitude of health workers</td></tr>
</table>

	**(For herbal drug sellers and traditional doctors)**	• Preference for traditional medicine<br>• Cultural norms and beliefs about seeking care in hospital<br>• Cultural norms and beliefs malaria<br>• Perceived seriousness of malaria<br>• Perceived threat of malaria<br>• Frequency of malaria episode
6	Let us briefly discuss how health seeking behaviour and source of care differ by socioeconomic group?	**Probe** for –where each of the following categories of people usually seek malaria care?<br>• Rich people in the community<br>• Poor people in the community<br>• People with formal education<br>• People without formal education<br>(**For each of the categories, probe for the common source of malaria treatment e.g** - government institutions, private clinics, pharmacy, chemists, drug peddlers/hawkers, traditional healing homes, herbal drug stores/kiosks
	**Malaria medications being used and their local names**	
7	a) What can you say about the malaria medications that community members use?	• Please tell us about malaria medications that are commonly used among adult population in the community.<br>○ **Probe for**, the local names for the malaria medications being used by adults in the community<br>• Tell us about the malaria medications that are commonly used among children in the community.<br>○ **Probe for**, the local names for the malaria medications being used by children in the community<br>• What are your views about the cost of the malaria medications (orthodox medicine)?<br>• How affordable do you think the cost of malaria medications is for community members?
	b) What can you say about the use of herbal drugs for treating malaria?<br><br>**(For herbal drug sellers and traditional doctors)**	• Types of herbal drugs used for treating malaria<br>○ **Probe for**, the local names for the herbal malaria drugs<br>• Perceived efficacy of the herbal drugs used for treating malaria<br>• How common the use of herbal malaria drugs is in the community<br>• Characteristics of community women who prefer to use herbal drugs for malaria treatment<br>• Reasons community members prefer to use herbal drugs for malaria treatment<br>• What are your views about the cost of the herbal malaria drugs?<br>• What determines the type of herbal malaria drugs that you give to your patients?

		• Under what condition should community members who use herbal malaria drugs seek healthcare in the hospital for malaria treatment?
	c)What can you say about your competency to treat and manage malaria infections?	**Probe** for the competency to manage:<br>• Malaria infections involving adults<br>• Malaria infections involving under-five children<br>• Malaria infection involving pregnant women<br>• Complicated malaria infections<br>• Individuals having frequent malaria episodes
	**Showing of pictures or physical packaging of malaria medications to improve recall and answers about usage of malaria medication**	
8	a) What are your opinions about the showing of pictures or physical packaging of malaria medications with the intention of improving recall and answers about usage of malaria medication?	**Probe** for<br>• Why showing of pictures or physical packaging of malaria medications can possibly improve recall and answers about usage of malaria medication?
	b) In your opinion what other ways or means do you think can help community members to improve recall and answer about usage of malaria medications?	
	**Factors influencing use of malaria medications and treating malaria at a hospital**	
9	a)Please share with us the things that you think do influence community members decisions about choice of malaria medications	**Probe** for<br>• **Individual factors that influence the decisions about what malaria medicine to use – e.g** Age, sex (male or female), marital status, economic status, being pregnant, medical history, perceived seriousness of malaria, perceived threat of malaria, preference for particular type of malaria medications (orthodox drugs), preference for herbal malaria drugs<br>• **Drug related factors that influence the decisions about what malaria medicine to use –** e.g Availability of drug in hospital or drug stores, number of days expected to use drugs, number of capsules/dosages, taste of drug, side effects of drugs, drug resistance, availability of herbal malaria drugs, preference for herbal malaria drugs, proliferation of fake malaria medicines, giving of genuine malaria medications<br>• **Social-cultural factors that influence the decisions about what malaria medicine to use –** e.g support from partners, support family members, support from

		friends/peers, media influence and drug adverts, cultural norms and values<br>• **Health system related factors that influence the decisions about what malaria medicine to use-** e.g availability of drugs in hospital/pharmacy, prescription of drug, advice from health workers<br>• **Policy related factors e.g** –governments and regulatory bodies recommendation relating to antimalarial drugs that should be first line of treatment<br>• **Disease pattern related factors e.g** –uncomplicated vs severe malaria<br>• **Economic related factors** e.g – cost of buying malaria medications, affordability of preferred malaria medications
	b)What is your view about the cost of treating malaria in the hospital?	• How affordable do you think the cost of treating malaria is for community members in the health facility?
	**Common methods that community members use to protect themselves from malaria**	
10	a) Please tell us about the common methods that people use to protect themselves from malaria in this community	**Probe** for (if not mentioned):<br>• Use of insecticide sprays<br>• Use of insecticides treated bed nets<br>• Use of mosquito replants<br>• Use of coils<br>• Use of window and door screens<br>• Wearing of long-sleeved clothing and long pants<br>• Malaria prophylaxis
	b) Which method is most commonly used by people to protect themselves from malaria?	**Probe** for<br>• Reasons for being the most commonly used
	c) Which methods do community members usually use to protect under-five children from malaria?	
	**Participation in community-based malaria programme**	
11	a) We are planning to put in place a free community-based programme where community members will be asked to consistently report malaria cases or symptoms through their cell phones. Our goal is to understand the transmission of malaria in the community to inform where interventions go.	

	What is your opinion about how community members will perceive the programme	
	b) What can facilitate the participation of community members in the programme?	
	c) Supposed we ask community members to be reporting malaria cases or symptoms via text message as part of our community-based malaria programme, what is your opinion about it?	**Probe** for:<br>• How easy do you think it will be for community members to participate?<br>• How willing do you think community members will be?<br>• What barriers do you think may be associated with it?<br>• What do you think we can encourage community members to be reporting consistently malaria cases or symptoms via text message?
	d) What is your opinion about asking community members to be reporting malaria cases or symptoms via a mobile application as part of our community-based malaria programme?	**Probe** for:<br>• How easy do you think it will be for community members to participate?<br>• How willing do you think community members will be?<br>• What barriers do you think may be associated with it?<br>• What do you think we can encourage community members to be reporting consistently malaria cases or symptoms via a mobile application?
	e) If we want community members to be reporting malaria cases or symptoms consistently what do you think would be the preferred way or means for this?	**Probe** for (if not mentioned):<br>• Face-to-face with a project volunteer<br>• Through community representative<br>• Through SMS<br>• Through WhatsApp<br>• Through mobile application<br>• Through hotline<br>• Through community meeting etc.
	**Conclusion and other relevant information**	
12	a) Please tell us about on-going or existing community-based malaria interventions in this community	
	b) What suggestions do you have about how malaria can be controlled in this community?	
	c) What other suggestions do you have that can help us with the community-based malaria programme that are planning?	

Socio-demographic information

Ward and LGA ……………………………… Sex: ……………………

Age in years (at last birthday) ……….. Highest level of Education……………..

Primary occupation …………………… Other occupation(s) …………………….

Number of years spent as informal healthcare provider……………………

Type of settlement ……………………………………..……………..

Name of Community/Area……………………………………..…………..

Appendix 4

Field assessment of the burden and determinants of malaria transmission in urban areas – key informant interview guide for community leaders

Who to interview: Community leaders- These include:

1. Opinion leaders
2. Traditional leaders
3. Religious leaders
4. Women leaders

Introduction
My name is …………..and my colleagues are………….. I am working with University of Ibadan and Bayero University Kano. We would like your opinion on various issues to enable us understand malaria transmission in urban areas. The information we collect will help the government to plan health services to prevent malaria infections by ensuring you receive suitable interventions. You have been specially invited to this key informant interview and we thank you for honouring our invitation.

Purpose
The purpose of this key informant interview is to investigate the following

1. How community members manage suspected malaria infections
2. Where community members seek care for malaria
3. Factors that influence care seeking for malaria in a hospital,
4. How health seeking behaviour and source of care differ by socioeconomic group
5. Local names for malaria medications
6. How showing of pictures or physical packaging of malaria medications can improve participants' recall and answers about usage of malaria medication
7. Factors that influence decisions about what malaria medicine to use
8. Common methods that people use to protect themselves from themselves from malaria
9. Understand facilitators and barriers to participating in a community-based disease reporting programme for malaria

The information learned in this key informant interview will be used to guide the development of other components of our study including surveys and longitudinal component that we are planning to conduct.

Procedure
The key informant interview, which will last for 40 – 60 minutes, will entail asking you some open-ended questions. In addition to the interview questions, we will be asking for some of your socio-demographic information. In the course of this interview, your views will be respected and will not be used against you in any way. This interview will be taped, so please speak up and speak clearly. We ask for your consent to record the interview so that we will not miss out anything from the information you will be providing to us through the interview. Please do share your views without mentioning people's names. We want the interview to be anonymous and to be confidential as possible. You can choose whether or not to participate in the interview, and you may stop at any time during the course of the study. There is no right or wrong view, so feel free to express yourself. Please note that your participation in this interview is voluntary. Your decision not to be involved or drop out at any point will not attract any penalty.

Benefits
Your participation in this study may not provide any personal benefit to you. However, should you decide to participate in this study, you will be doing society a great service because the findings of this study will be useful in the design of interventions and programmes for the control and prevention of malaria.

Risks
There are no known or anticipated risks associated with participation in this study beyond those experienced during an average conversation. If a question, or the discussion, makes you uncomfortable, you can choose not to answer.

Confidentiality
The information you share will be kept confidential. Identifying information will be removed from the transcripts. The transcripts and other electronic data will be retained for a maximum of 5 years, after which they will be destroyed. Data will be stored in an encrypted folder on protected laptop. Only the research team will have access to study data. No identifying information will be used in any presentations or publications based on this research.

Contact
If you have any questions or concerns regarding this study, please contact:
Professor IkeOluwapo Ajayi
Email: ikeyajayi2003@yahoo.com, Tel: 08023268431
Director, Institute for Advanced Medical Research and Training (IMARAT),
College of Medicine, University of Ibadan

Thank you for choosing to participate in the study. Kindly show by using any of the following 2 boxes, that your participation in this study was voluntary.

☐ I will participate

☐ I will not participate

S/N	Main questions	Follow up questions or hints
	**Introduction and general questions**	
1	a) What can you say about the living conditions of community members?	• Financial condition or status of community members<br>o (**Probe for**, opinions about categorization of community members based on economic or financial status)<br>• Health facilities available in the communities<br>o **Probe for**, types of health facilities (private hospital, primary health facilities, secondary health facilities, tertiary health facilities)<br>o **Probe for**, the most preferred by community members (private hospital, primary health facilities, secondary health facilities, tertiary health facilities)
	b) Where does the community members generally prefer to go for health care?	**Probe** -(if not mentioned):<br>• Health facilities (private hospitals, primary health facility, secondary health facilities, tertiary health facilities).<br>• Patent medicine stores<br>• Traditional healing home<br>• Drug peddlers e.t.c
	c) In this community if someone is pregnant, where would they typically go for antenatal care?	**Probe** for the following (if not mentioned):<br>• Health facilities (private hospitals, primary health facility, secondary health facilities, tertiary health facilities).<br>• Traditional Birth Attendants<br>• Traditional doctors<br>• Faith-based maternity homes<br>• No where/prefer to give birth at home<br>o Opinions on why some pregnant women prefer not to attend antenatal care in the hospitals
	d) What are the common diseases in this community	• Which diseases are most common among under-five children?<br>• Which diseases are most common among adult population?<br>• Which diseases are the most severe diseases?
	**Basic understanding of malaria**	
2	Now let us discuss specifically on some basic issues relating to malaria.<br><br>a) How common is malaria in this community?	What categories of persons are most affected by malaria?<br>Probe for:<br>o Under-five children<br>o Aged people<br>o Pregnant women<br>o People with sickle cell anemia e.t.c
	b) How do community members usually get to know that they have malaria?	**Probe** for the following (if not mentioned):<br>o Through observation of signs and symptoms that are suggestive of malaria<br>o Clinical examination

		○ Lab examination
	c) In your opinion, what are the signs and symptoms of malaria	
	**Practices relating to management of malaria in the community**	
3	a) How do adult members in community manage suspected malaria infections?	**Probe** for the following (if not mentioned):<br>○ Home-based care<br>○ Self-medication<br>○ Use of herbs<br>○ Use of drugs or medicines with prescription
	b) Tell us about how suspected malaria infections of under-five children are being managed in the community?	**Probe** for the following (if not mentioned):<br>○ Home-based care<br>○ Self-medication<br>○ Use of herbs<br>○ Use of drugs or medicines with prescription
	c) Tell us about how suspected malaria infections in adolescents are being managed in the community	**Probe for the following (if not mentioned):**<br>○ Home-based care<br>○ Self-medication<br>○ Use of herbs<br>Use of drugs or medicines with prescription
	d) What efforts are being put in place by the community relating to the prevention and control of malaria?	
	**Malaria related health-seeking behaviours of community members**	
4	a) Where do community members seek care for malaria?	**Probe** for the following (if not mentioned)<br>○ Traditional healing homes<br>○ Patient Medicine Vendors stores<br>○ Drug hawkers/peddlers<br>○ Pharmacy stores<br>○ Herbal drug stores/kiosks<br>○ Hospitals (Private hospital, government hospital)<br>**Probe for** where community members seek care for malaria the most and reasons
5	b) What do you think usually influence community members to seek malaria in a hospital?	**Probe** for the following<br>• Money for paying bills for treatment<br>• Money for transportation to hospital<br>• Distance of hospital<br>• Access to hospital<br>• Availability of hospital

		• Attitude of health workers<br>• Preference for traditional medicine<br>• Preference for a particular type of hospital (Private hospital, primary health centre, secondary health care facility)<br>• Cultural norms and beliefs about seeking care in hospital<br>• Cultural norms and beliefs malaria<br>• Perceived seriousness of malaria<br>• Perceived threat of malaria<br>• Frequency of malaria episode
6	Let us briefly discuss how health seeking behaviour and source of care differ by socioeconomic group	**Probe** for –where each of the following categories of people usually seek malaria care?<br>• Rich people in the community<br>• Poor people in the community<br>• People with formal education<br>• People without formal education<br><br>(**For each of the categories, probe for the common source of malaria treatment e.g** - government institutions, private clinics, pharmacy, chemists, drug peddlers/hawkers, traditional healing homes, herbal drug stores/kiosks)
	**Malaria medications being used and their local names**	
7	a) What can you say about the malaria medications that community members use?	• Please tell us about malaria medications that are commonly used among adult population in the community.<br>○ **Probe for**, the local names for the malaria medications being used by adults in the community<br>• Tell us about the malaria medications that are commonly used among children in the community.<br>○ **Probe for**, the local names for the malaria medications being used by children in the community<br>• What are your views about the cost of the malaria medications (orthodox medicine)?<br>• How affordable do you think the cost of malaria medications is for community members?
	b) Please think about it If you were sick, which malaria medications would you take	**Probe for**,<br>• The local names for the malaria medications<br>• The malaria medication(s) used the last time participant had malaria infection

	c) What can you say about the use of herbal drugs for treating malaria by community members?	- Types of herbal drugs used for treating malaria **Probe for**, the local names for the herbal malaria drugs - Perceived efficacy of the herbal drugs used for treating malaria - How common the use of herbal malaria drugs is in the community - Characteristics of community women who prefer to use herbal drugs for malaria treatment - Reasons community members prefer to use herbal drugs for malaria treatment - What are your views about the cost of the herbal malaria drugs? - Under what condition should community members who use herbal malaria drugs seek healthcare in the hospital for malaria treatment?
	**Showing of pictures or physical packaging of malaria medications to improve recall and answers about usage of malaria medication**	
8	a) What are your opinions about the showing of pictures or physical packaging of malaria medications with the intention of improving recall and answers about usage of malaria medication?	**Probe** for Why showing of pictures or physical packaging of malaria medications can possibly improve recall and answers about usage of malaria medication?
	b) In your opinion what other ways or means do you think can help community members to improve recall and answer about usage of malaria medications?	
	**Factors influencing use of malaria medications and treating malaria at a hospital**	
9	a) Please share with us the things that you think do influence community members decisions about choice of malaria medications	**Probe** for **Individual factors that influence the decisions about what malaria medicine to use – e.g** Age, sex (male or female), marital status, economic status, being pregnant, medical history, perceived seriousness of malaria, perceived threat of malaria, preference for particular type of malaria medications (orthodox drugs), preference for herbal malaria drugs **Drug related factors that influence the decisions about what malaria medicine to use –** e.g Availability of drug in hospital or drug stores, number of days expected to use drugs, number of capsules/dosages, taste of drug, side effects of drugs, drug resistance, availability of herbal malaria drugs, preference for herbal malaria

		drugs, proliferation of fake malaria medicines, giving of genuine malaria medications<br>• **Social-cultural factors that influence the decisions about what malaria medicine to use –** e.g support from partners, support family members, support from friends/peers, media influence and drug adverts, cultural norms and values<br>• **Health system related factors that influence the decisions about what malaria medicine to use-** e.g availability of drugs in hospital/pharmacy, prescription of drug, advice from health workers<br>• **Policy related factors e.g** –governments and regulatory bodies recommendation relating to antimalarial drugs that should be first line of treatment<br>• **Disease pattern related factors e.g** –uncomplicated vs severe malaria<br>• **Economic related factors** e.g – cost of buying malaria medications, affordability of preferred malaria medications
	b) What is your view about the cost of treating malaria in the hospital?	• How affordable do you think the cost of treating malaria is for community members in the health facility?
	**Common methods that community members use to protect themselves from malaria**	
10	a) Please tell us about the common methods that people use to protect themselves from malaria in this community	**Probe** for (if not mentioned):<br>• Use of insecticide sprays<br>• Use of insecticides treated bed nets<br>• Use of mosquito replants<br>• Use of coils<br>• Use of window and door screens<br>• Wearing of long-sleeved clothing and long pants<br>• Malaria prophylaxis
	b) Which method is most commonly used by people to protect themselves from malaria?	**Probe** for<br>• Reasons for being the most commonly used
	**c)** Which methods do community members usually use to protect under-five children from malaria?	
	**Participation in community-based malaria programme**	
11	a) We are planning to put in place a free community-based programme where community members will be asked to consistently report malaria cases or symptoms	

	through their cell phones. Our goal is to understand the transmission of malaria in the community to inform where interventions go. What is your opinion about how community members will perceive the programme. What is your opinion about how community members will perceive the programme?	
	b) What can facilitate the participation of community members in the programme?	
	c) Supposed we ask community members to be reporting malaria cases or symptoms via text message as part of our community-based malaria programme, what is your opinion about it?	**Probe** for:<br>• How easy do you think it will be for community members to participate?<br>• How willing do you think community members will be?<br>• What barriers do you think may be associated with it?<br>• What do you think we can encourage community members to be reporting consistently malaria cases or symptoms via text message?
	d) What is your opinion about asking community members to be reporting malaria cases or symptoms via a mobile application as part of our community-based malaria programme?	**Probe** for:<br>• How easy do you think it will be for community members to participate?<br>• How willing do you think community members will be?<br>• What barriers do you think may be associated with it?<br>• What do you think we can encourage community members to be reporting consistently malaria cases or symptoms via a mobile application?
	e) If we want community members to be reporting malaria cases or symptoms consistently what do you think would be the preferred way or means for this?	**Probe** for (if not mentioned):<br>• Face-to-face with a project volunteer<br>• Through community representative<br>• Through SMS<br>• Through WhatsApp<br>• Through mobile application<br>• Through hotline<br>• Through community meeting etc.
	**Conclusion and other relevant information**	
12	a) Please tell us about on-going or existing community-based malaria interventions in this community	

	b) What suggestions do you have about how malaria can be controlled in this community?	
	c) What other suggestions do you have that can help us with the community-based malaria programme that are planning?	

Socio-demographic information

Ward and LGA …………………………… Sex: ……………………

Age in years (at last birthday) ……….. Highest level of Education……………..

Primary occupation …………………… Other occupation(s) ……………………..

Position…………………………………

Type of settlement ……………………………………………………..

Name of Community/Area……………………………………………..
